# A Cold-Active Protease Tissue Dissociation Protocol for the Preservation of the Tendon Fibroblast Transcriptome

**DOI:** 10.21769/BioProtoc.5293

**Published:** 2025-05-05

**Authors:** Arul Subramanian, Pavan K. Nayak, Thomas F. Schilling

**Affiliations:** Department of Developmental and Cell Biology, University of California, Irvine, CA, USA

**Keywords:** Tendon fibroblast, Tenocyte, *Bacillus licheniformis*, RNA sequencing, Tissue dissociation

## Abstract

Traditional tissue dissociation methods for bulk- and single-cell sequencing use various protease and/or collagenase combinations at temperatures ranging from 28 to 37 °C, which cause transcriptional cell stress that may alter data interpretation. Such artifacts can be reduced by dissociating cells in cold-active proteases, but few studies have shown that this improves cell-type specific transcription, particularly in tissues hypersensitive to mechanical integrity and extracellular matrix (ECM) interactions. To address this, we have dissociated zebrafish tendons and ligaments in subtilisin A at 4 °C and compared the results with 37 °C collagenase dissociation using bulk RNA sequencing. We find that high-temperature collagenase dissociation causes general cell stress in tendon fibroblasts (tenocytes) as reported in previous studies with other cell types, but also that high temperature specifically downregulates hallmark genes involved in tenocyte specification and ECM production in vivo. Our results suggest that cold-protease dissociation reduces transcriptional artifacts and increases the robustness of RNA-sequencing datasets such that they better reflect native in vivo tissue microenvironments.

Key features

• Utilizing a cold-active protease derived from the Himalayan soil bacterium *B. licheniformis* for tissue dissociation preserves cell transcriptomes, increasing data quality of downstream sequencing experiments.

• This method is reproducible and requires no extra equipment for tissue agitation.

• Tenocytes isolated using this method show lower stress and better preserved native expression of key tenocyte markers and ECM genes than with traditional warm-dissociation methods.

• This protocol is ideal for cell types that are particularly sensitive to microenvironment signals or are embedded in extracellular matrix.

## Graphical overview


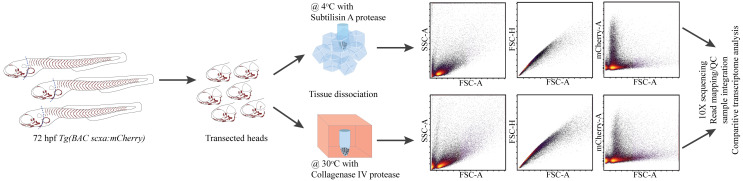


## Background

Standard protocols for sequencing assays from organs and tissues such as bulk or single-cell RNA sequencing (RNA-seq) generally start with tissue dissociation, often followed by fluorescence-activated cell sorting (FACS) to isolate cell populations of interest. Whereas most dissociation protocols utilize proteases such as pronase, trypsin, and collagenase at ~28–37 °C, RNA-seq assays in many different cell types and organisms have shown cell stress–associated gene expression, likely artifacts of dissociation that suggest a need for reinterpretations of many published sequencing datasets [1–4]. Alternatively, tissues can be dissociated at 6 °C or lower using the protease subtilisin A, derived from the Himalayan soil bacterium *Bacillus licheniformis*. This dramatically reduces many of these stress-related expression signatures, presumably by preserving active cellular transcription machinery [2,3,5–7]. It also remains unclear whether traditional high-temperature dissociation protocols specifically affect cell types sensitive to microenvironmental cues. For example, tissue-resident fibroblasts in tendons and ligaments (i.e., tenocytes and ligamentocytes) secrete and become embedded within surrounding ECM. Tendons attach muscles to bone and are exposed to the constant load of muscle contraction. Therefore, in order to adapt to this load, tenocytes must tightly control their transcriptional responses to ECM signals and secretion of structural ECM genes, making them highly susceptible to environmental changes during cell dissociation [8,9].

RNA-seq studies of musculoskeletal tissues have dissociated cells at 28.5–37 °C [10–12]. Since dissociation artifacts in single-cell RNA-seq (scRNA-seq) experiments can lead to aberrant unsupervised cell clustering, these stand to benefit the most from cold protease approaches [1]. Here, we present a modified subtilisin A protease protocol for the dissociation of tendon tissue on ice and compare its effectiveness with existing collagenase-based dissociation protocols. We dissociate cells from dissected heads of *Tg(scx:mCherry)* (marking tenocytes) zebrafish embryos at 72 h post-fertilization (hpf) at either ~4 or ~28.5 °C, suspend cells for FACS isolation, and perform RNA-seq of mCherry+ cranial tenocytes. Cold dissociation decreases the expression of cell stress and preserves the expression of ECM genes. These results suggest that traditional high-temperature dissociation protocols stress cells and alter ECM expression differentially in connective tissue cell types that heavily depend on their native in vivo context.

## Materials and reagents

**Biological materials**

*Note: The cold protease dissociation assay is not specific for zebrafish or tendons.*

1. Live *Danio rerio* (zebrafish) embryos of appropriate developmental stage (RRID: NCBITaxon_7955)

**Reagents**

1. PBS (Gibco, catalog number: 10-010-031)

2. DPBS (Gibco, catalog number: 14190-144)

3. Protease from *Bacillus licheniformis* (Sigma-Aldrich, catalog number: P5380, UniProt ID P00780)

4. DNase I (Roche, catalog number: 11284932001, UniProt ID P00639)

5. Ethylenediaminetetraacetic acid (EDTA) (Sigma-Aldrich, catalog number: 431788, CID 6049)

6. Calcium chloride dihydrate (CaCl_2_·2H_2_O) (Sigma-Aldrich, catalog number: C3306, CID 24844)

7. Potassium chloride (KCl) (Sigma-Aldrich, catalog number: P3911, CID 4873)

8. Sodium chloride (NaCl) (Fischer Scientific, catalog number: S271-1, CID 5234)

9. HEPES [4-(2-hydroxyethyl)-1-piperazineethanesulfonic acid] (Sigma-Aldrich, catalog number: 54457, CID 23831)

10. 1 M Tris-HCl, pH 9.0 (Alkali Scientific, catalog number: DB0227, CID 93573)

11. Ethyl 3-aminobenzoate methanesulfonate (MS-222/Tricaine) (Millipore Sigma, catalog number: A5040, CID 261501)

12. Trypan blue 0.4% (Gibco, catalog number: 15250061, CID 135442966)

13. Krayden Dow Sylgard 184 Silicone Elastomer Kit, clear, 1.1 lb (Fisher Scientific, catalog number: NC9285739)

14. Bovine serum albumin (BSA) (Sigma-Aldrich, catalog number: A7030, UniProt ID P02769)

**Solutions**

1. Ringers solution (see Recipes)

2. MS-222/Tricaine solution (see Recipes)

3. MS-222/Tricaine working solution (see Recipes)

4. Cold protease stock solution (see Recipes)

5. DNAse stock solution (see Recipes)

6. Cold protease working solution (see Recipes)

7. DPBS-BSA solution (see Recipes)

8. Silicone polymer mix (see Recipes)

**Recipes**

**1. Ringers solution [13]**

**Table:** 

Reagent	Final concentration	Volume
5 M NaCl	116 mM	11.6 mL
250 mM KCl	2.9 mM	5.8 mL
1 M CaCl_2_·2H_2_O	10 mM	5 mL
1 M HEPES pH 7.2	5 mM	2.5 mL

Adjust pH to 7.2 using NaOH, then add water to 500 mL.

Can be stored at 4 °C for up to three months.

**2. MS-222/Tricaine solution [13]**

**Table:** 

Reagent	Final concentration	Volume
MS-222/Tricaine	15.3 mM	400 mg
1 M Tris-HCl, pH 9.0	5.25 mM	2.1 mL

Adjust pH to 7.2 using NaOH, then add water to 400 mL.

Can be stored at 4 °C for up to three months.

**3. MS-222/Tricaine working solution [13]**

**Table:** 

Reagent	Final Concentration	Volume
MS-222/Tricaine	15.3 mM	4.2 mL
Ringer’s solution		Up to 100 mL

**4. Cold protease stock solution**

**Table:** 

Reagent	Final concentration	Volume
Protease from *B. licheniformis*	100 mg/mL	100 mg
1× PBS (no calcium, no magnesium)	1×	800 μL

Dissolve by repeated pipetting, add 1× PBS to 1,000 μL.

Prepare 100 mL aliquots in cryostorage tubes and store at -80 °C.

**5. DNase stock solution**

**Table:** 

Reagent	Final concentration	Volume
DNase I	20 U/μL	10 mg
1× PBS (no calcium, no magnesium)	1×	800 μL

Dissolve by repeated pipetting, add 1× PBS to 1,000 μL.

Prepare 100 mL aliquots in microcentrifuge tubes and store at -20 °C.

**6. Cold protease working solution**

**Table:** 

Reagent	Final concentration	Volume
100 mg/mL cold protease stock solution	10 mg/mL	100 μL
1 M CaCl_2_	5 mM	5 μL
0.5 M EDTA	0.5 mM	1 μL
20 U/μL DNAse stock solution	100 U/mL	5 μL

Dissolve by repeated pipetting, add 1× DPBS to 1,000 μL.

Place on ice bath (4 °C) until use.

**7. DPBS-BSA solution**

**Table:** 

Reagent	Final concentration	Volume
BSA	0.01%	0.5 mg
1× DPBS (no calcium, no magnesium)	1×	4.5 mL

Dissolve by repeated pipetting, add 1× DPBS to 5 mL.

Place on ice bath (4 °C) until use.

**8. Silicone polymer solution**

**Table:** 

Reagent	Final concentration	Volume
SYLGARD^TM^ 184 Silicone elastomer base	91%	40 mL
SYLGARD^TM^ 184 Silicone curing agent	9%	4 mL

Mix by inversion on a nutator.

Store the mixed solution at -20 °C in an upright position to allow bubbles to rise up for a minimum of 24 h. After 24 h, the solution can be poured into 60 × 15 mm Petri dishes to the required height. Place the dishes in a 37 °C incubator for at least 12 h to set. The set plates can be stored at room temperature until use.

**Laboratory supplies**

1. 24-well tissue culture plates, flat bottom, ultra-low attachment (Costar, catalog number: 3473)

2. Micropipettes and low retention tips

3. 21 G PrecisionGlide needles (BD, catalog number: 305165)

4. 1 mL insulin syringe (BD, catalog number: 329654)

5. 40 μm cell strainer (Pluriselect, catalog number: 43-10040-40)

6. Glass slides, 22 × 75 × 1 mm (Fisherbrand, catalog number: 12-550-A3)

7. Coverslips, 22 × 22 × 0.13 mm (Fisherbrand, catalog number: 12-542-B)

8. Transfer pipettes (Fisherbrand, catalog number: 13-711-7M)

9. Ice bath

## Equipment

1. Refrigerated centrifuge (Eppendorf, model: 5430R)

2. Fixed angle rotor for 15 mL tubes (Eppendorf, model: F-35-6-30)

3. Dissecting scope (Zeiss, model: Stemi 2000 C)

4. Compound microscope with 40× objective (Zeiss, model: Axioplan 2)

## Procedure

**A. Preparation of zebrafish heads for dissociation**

**Figure 1.**
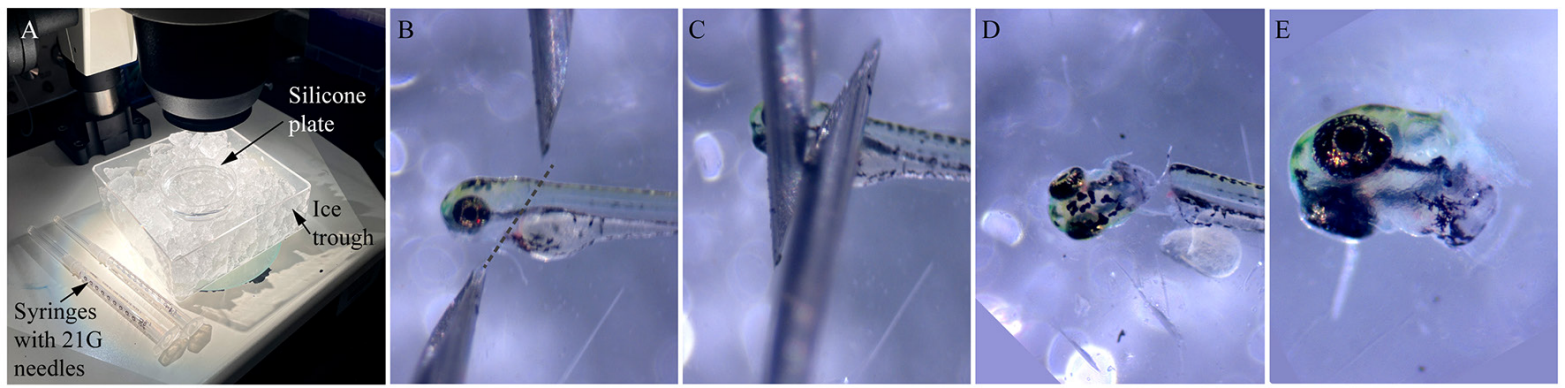
Stages of zebrafish embryo head dissection. (A) Ice trough with silicone plate containing ice-cold Ringer’s solution and syringes with unused 21 G needles. (B–E) Snapshots showing the position of the needles (dotted line shows the direction of the cut) in relation to a 72 h postfertilization (hpf) embryo to perform a quick and clean separation of the head from the trunk.

1. Use a pipette to transfer zebrafish embryos (N = 50) (72 h post-fertilization) to a silicone plate containing MS-222/Tricaine working solution on an ice bath.

2. Attach two 1 mL syringes with their plungers removed to 21 G needles.

3. Place the needles at a 45° angle dorsal and ventral to the heads of the embryos and just posterior to the pectoral fin, with the sharper edge facing the silicone surface (Figure 1B).

4. Rapidly drag the two needles simultaneously across the embryo in a scissor-like motion to sever the head from the trunk (Figure 1C, D).

**B. Tissue dissociation using cold protease**

**Figure 2.**
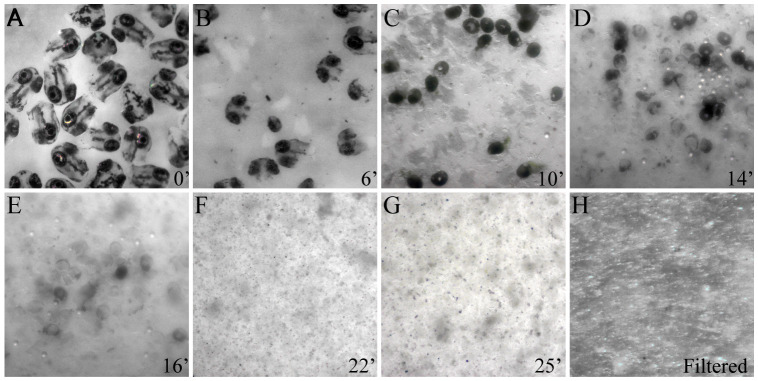
Stages of tissue dissociation of zebrafish embryonic heads. (A–G) Snapshots of heads removed from 72 h postfertilization (hpf) zebrafish embryos showing progressive tissue dissociation at 4 °C by subtilisin A activity. (H) Cell suspension post-dissociation after filtration using a 40 μm filter to remove cell debris and clumps.

1. Transfer heads to a well of a 24-well tissue culture plate (Figure 2A).

2. Gently aspirate the Ringer’s solution and replace it with 1 mL of cold protease working solution. Gently aspirate and expel the contents into a 1 mL pipette tip using a micropipette twice and place the plate on an ice bath.

3. Triturate the tissue dissociation mixture, aspirating and expelling it multiple times, for 15 s with a 1 mL pipette tip using a micropipette once every 2 min (Figure 2B–G).

4. Observe the dissociation mixture under a dissecting microscope to ensure complete tissue dissociation (Figure 2F, G). The total time for complete tissue dissociation depends on the embryonic stage and the complexity of ECM composition. Dissociation of 72 hpf embryonic heads was completed in ~25 min using freshly prepared enzyme aliquots.

**C. Preparation of single-cell suspension**

1. Position a 40 μm cell strainer over a 15 mL tube on an ice bath.

2. Transfer the tissue dissociation mixture to the cell strainer and gently tap the strainer to break up undissociated clumps of cells in the mixture and get them through the filter (Figure 2H).

3. Add 9 mL of ice-cold 1× DPBS through the cell strainer to wash it, dilute the cell suspension, and stop protease activity.

4. Pellet the cells from the suspension at 4 °C using a refrigerated centrifuge at 600× *g* for 5 min.

5. Discard the supernatant and resuspend the pellet in 1 mL of ice-cold DPBS-BSA solution by trituration using a 1 mL tip and a micropipette.

**D. Validation of single-cell suspension viability**

1. Mix 2 μL of 0.4% trypan blue and 2 μL of cell suspension. Incubate the solution in an ice bath for 2 min.

2. Transfer 2 μL of the mixture to a slide, place a coverslip over the slide, and visualize under a compound microscope.

3. Confirm cell viability by trypan blue exclusion.

4. After confirming cell viability, the cell suspension can be used for downstream processing such as FACS and RNA-seq.

## Data analysis

**RNA sequencing**

Dissociated cell suspensions from cold and warm conditions were sorted on a Bio-Rad FACS Aria Fusion cell sorter located at the UCI Institute for Immunology Flow Cytometry Facility. Sorted mCherry+ cell suspensions were provided to the UCI Genomics High Throughput Facility (GHTF) for 10× library preparation and sequencing for ~500 M total reads using 3’ v3 chemistry.

**Processing of raw reads**

FASTQ reads for both cold and warm conditions obtained from GHTF were mapped to zebrafish genome version GRCz11 using CellRanger (version 3.1.0) [14] after modifying the genome and Genome Transfer Format (GTF) annotations with the addition of the *mCherry* nucleotide sequence (708 nucleotides). Web summary metrics post-alignment are shown in Table 1.

**Table 1.** CellRanger mapping quality metrics

Metrics	Cold condition	Warm condition
Estimated number of cells	14,531	21,755
Mean reads per cell	37,963	28,890
Median genes per cell	1,960	1,623
Number of reads	551,646,943	628,508,698
Valid Barcodes	97.8%	97.8%
Valid UMIs	100%	100%
Sequencing saturation	54.2%	45.4%
Reads mapped to genome	93.1%	93.7%
Reads mapped confidently to genome	78.6%	78.3%
Reads mapped confidently to transcriptome	61.9%	61.3%

**RNAseq QC and analysis in Seurat**

Filtered count matrices for each condition were converted into Seurat objects (version 4.0.5, R version 4.0.2) [15]. mCherry+ cells were kept for downstream analysis if they met the quality control criteria of 200 > genes/cell (nFeatures) < 4000 and mitochondrial gene expression < 5%. For anchoring/sample integration, individual Seurat objects were merged together with the *merge* function and data were normalized using *NormalizeData* function with default parameters (normalization.method = “LogNormalize”, scale.factor = 10000). Feature selection was carried out with the *FindVariableFeatures* function with default parameters (selection.method = “vst”, nfeatures = 2000). Data were scaled with *ScaleData()* function, and PCA was performed with *RunPCA* function with npcs = 30. All 30 PCs were used for UMAP reduction using *RunUMAP()* and nearest neighbor graph construction using *FindNeighbors().* Unsupervised clustering was performed with the *FindClusters* function using a resolution parameter of resolution = 0.5. Pseudo-bulk differential expression between warm and cold conditions was performed by switching identities of the integrated cold/warm dataset to the sample identities [Idents(Seurat_object) <- “orig.ident”] and using the *FindMarkers()* function with parameters min.pct = 0.1 and logfc.threshold = 0.1, with the default hypothesis test method using Wilcoxon Rank Sum test and Bonferroni correction for multiple hypothesis tests. Gene module aggregate scoring was performed by using the Seurat *AddModuleScore()* function with the default settings (nbin = 24 and ctrl = 100) on the integrated/anchored dataset. Gene lists for the fibrillar collagen module include *col1a1a, col1a1b, col1a2, col2a1a, col2a1b, col5a1, col5a2a, col5a2b, col5a3a, col5a3b*, and *col27a1b* and FACIT collagen module include *col9a1a, col9a1b, col9a2, col9a3, col12a1a, col12a1b, col14a1a*, and *col14a1b*. Statistical testing for expression differences displayed on all violin plots was performed with the *stat_compare_means()* function utilizing the default Wilcoxon Rank Sum test from the ggpubr package (version 0.4.0). The volcano plot was produced using the EnhancedVolcano package (version 1.8.0) with a p-value line drawn at 0.05 (pCutoff = 0.05) and fold change line drawn with FCcutoff = 0.5.

**Gene ontology analysis**

Gene ontology (GO) analysis was performed using the *enrichGO()* function from the clusterProfiler package (version 4.4.4) [16] with zebrafish genome annotations provided by the org.Dr.eg.db package (version 3.12.0), Biological Process (BP) GO category annotation, pvalueCutoff = 0.01, qvalueCutoff = 0.05, and Benjamini–Hochberg correction using pAdjustMethod = “BH”.

**Stress gene Venn diagram overlap analysis**

Comparisons of cold/warm datasets with existing published datasets were performed using (if comparing with a non-zebrafish organism dataset) gProfiler [17] to obtain orthologous human and mouse genes from our zebrafish differentially expressed gene (DEG) lists. Genes from the DEG list from cold/warm were then compared against the stress gene sets published by O’Flanagan et al. [3], Adam et al. [2], and van den Brink et al. [1] using the VennDiagram package (version 1.7.3) [18]. Overlap testing was performed with Fisher’s exact test using the GeneOverlap (version 1.26.0) [19] package with the number of coding genes for each genome placed in the genome.size argument as 25,592 for zebrafish (assembly GRCz11), 19,868 for human (assembly GRCh38.p14), and 22,011 for mouse (assembly GRCm39).

## Validation of protocol

**Differential tendon gene expression in cranial tenocytes dissociated with cold vs. warm protease**

**Figure 3.**
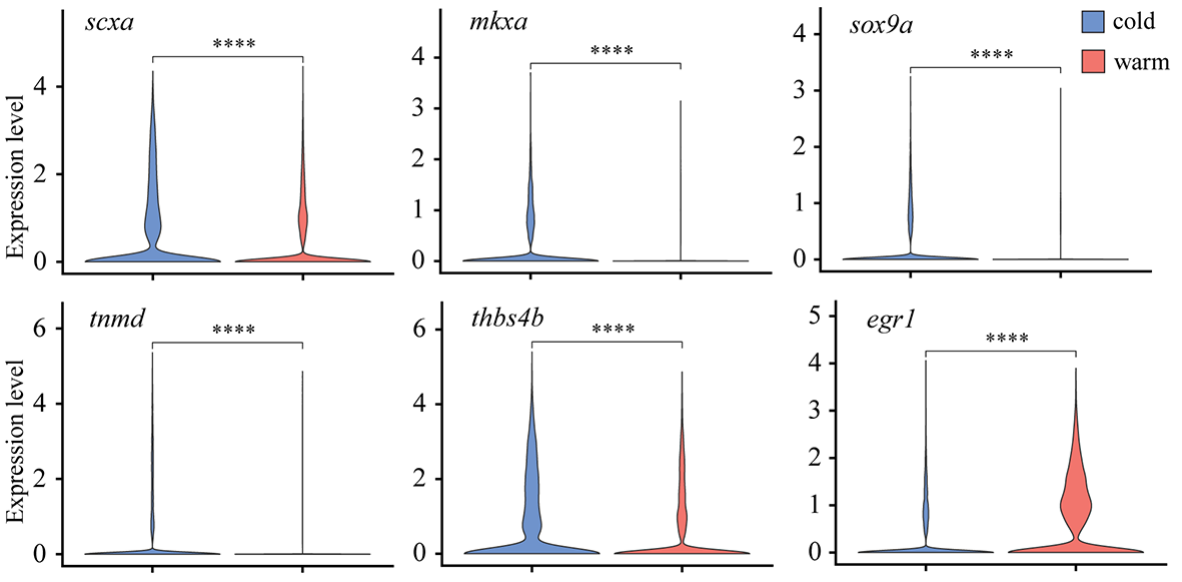
Differential expression of tenocyte marker genes between warm vs. cold dissociation conditions. Violin plots of tenocyte marker genes *scxa, mkxa, tnmd, thbs4b*, and *sox9a* all show significant downregulation, whereas *egr1* displays upregulation in warm dissociation conditions.

**Figure 4.**
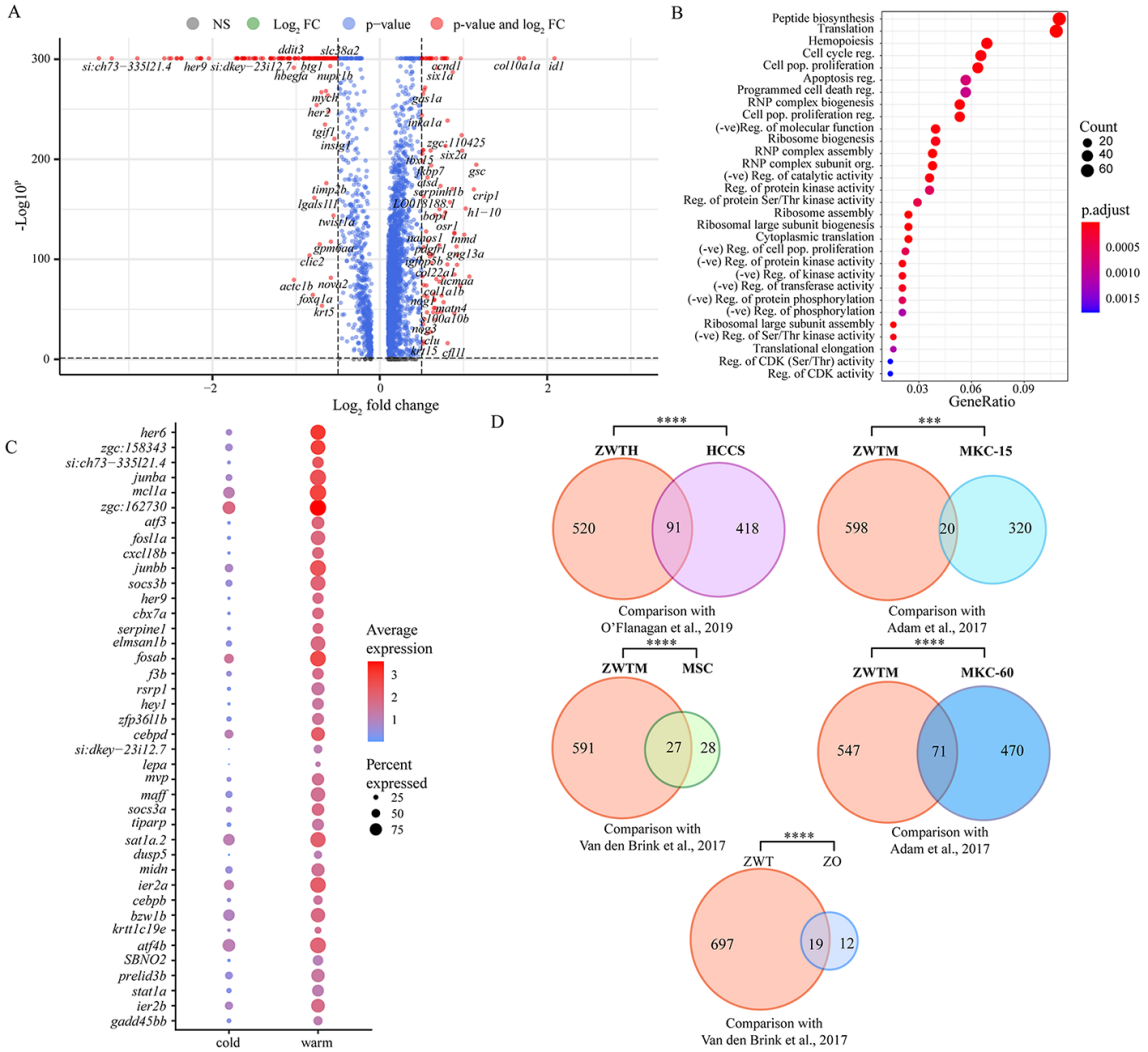
Pseudo-bulk differential gene expression analysis shows upregulation of stress markers under warm dissociation. (A) Volcano plot of pseudo-bulk differential expression analysis between cold and warm dissociation conditions showing genes downregulated (Log_2_-fold change above 0) and upregulated (Log_2_-fold change below 0) in warm conditions. Red points are genes with Log_2_-fold change > 0.5 or < -0.5 with adjusted p-value < 0.05. (B) Dot plot showing gene ontology (GO) term analysis for biological process (BP) terms of all significant warm-upregulated genes. (C) Top 40 significant genes (adjusted p-value < 0.05) upregulated in warm conditions, ordered by Log_2_-fold change. (D) Venn diagrams showing gene set overlaps between warm-upregulated genes with published dissociation cell-stress gene sets. ZWTH: zebrafish warm-upregulated tenocyte significant differentially expressed genes (DEG) converted to human orthologs; HCCS: human cancer cell stress core gene set published by O’Flanagan et al. [3]; ZWTM: zebrafish warm-upregulated tenocytes DEG converted to mouse orthologs; MSC: mouse muscle satellite cells stress-related gene set published by van den Brink et al. [1]; MKC-15: mouse kidney cells from 15 min dissociation published by Adam et al. [2]; MKC-60: mouse kidney cells from 60 min dissociation published by Adam et al. [2]; ZWT: zebrafish warm-upregulated tenocytes; ZO: zebrafish osteoblast cell stress-related gene set published by van den Brink et al. [1]. ns = not significant, * p < 0.05, ** p < 0.01, *** p < 0.001, **** p < 0.0001.

**Figure 5.**
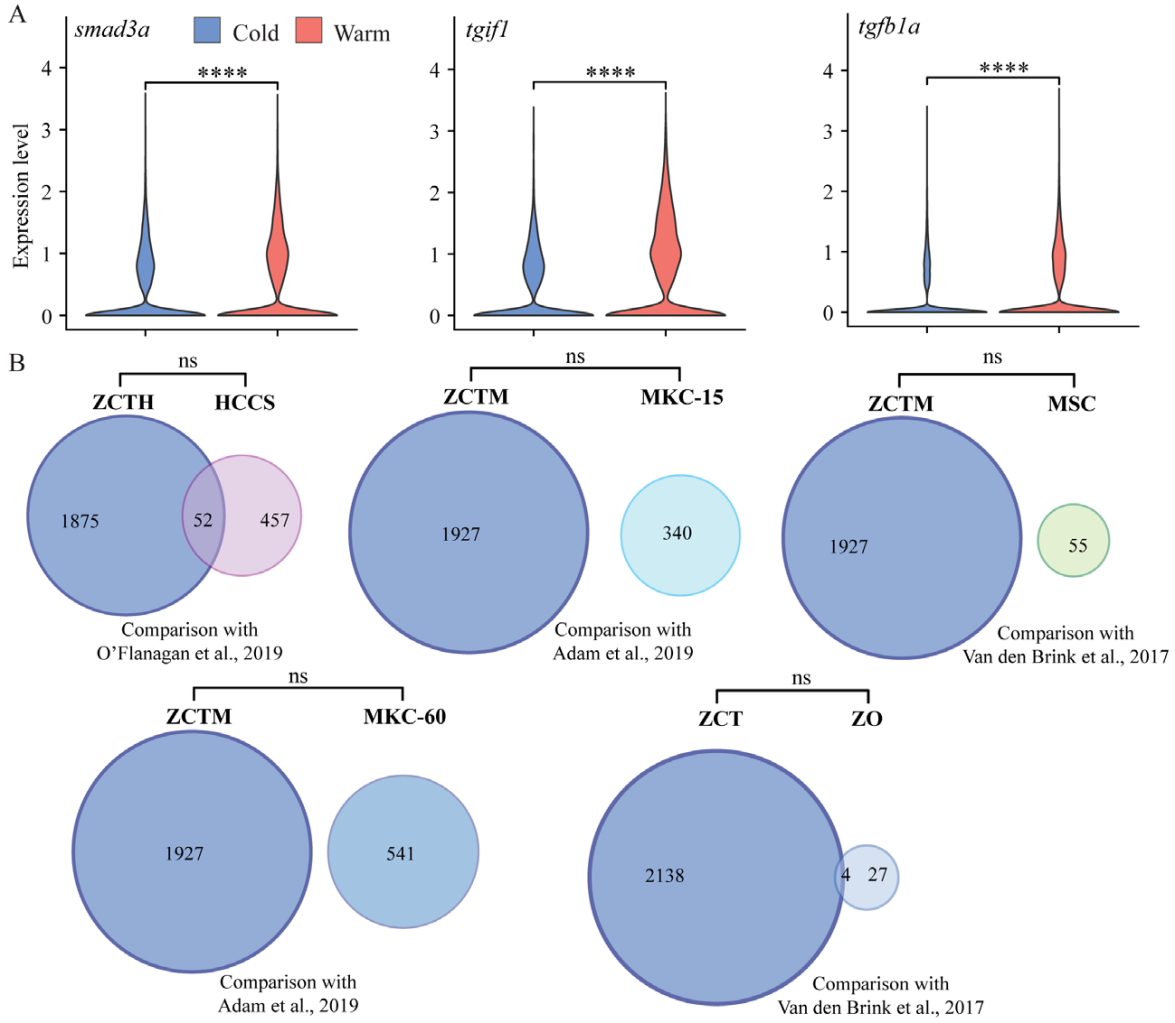
TGFβ marker expression in cold vs. warm dissociation conditions and cold upregulated genes compared with published cell-stress gene sets. (A) Violin plots showing relative expression of TGFβ signaling pathway genes *smad3a, tgif1*, and *tgfb1a*, all showing significant upregulation in warm dissociation conditions. (B) Venn diagrams showing gene set overlaps between cold-expressed genes with published dissociation cell-stress gene sets. ZCTH: zebrafish cold-upregulated significant differentially expressed genes (DEG) in tenocytes, converted to human orthologs; HCCS: human cancer cell stress core gene set published by O’Flanagan et al. [3]; ZCTM: zebrafish cold-upregulated tenocyte DEGs converted to mouse orthologs; MSC: mouse muscle satellite cells stress-related gene set published by van den Brink et al. [1]; MKC-15: mouse kidney cells from 15 min dissociation published by Adam et al. [2]; MKC-60: mouse kidney cells from 60 min dissociation published by Adam et al. [2]; ZCT: zebrafish cold-upregulated DEGs in tenocytes; ZO: zebrafish osteoblast cell stress-related gene set published by van den Brink et al. [1].

We first examined expression differences in hallmark marker genes crucial for proper tendon development and maturation by performing pseudo-bulk differential expression analysis, whereby all cells were integrated, clustered by condition (e.g., cold or warm dissociation), and the conditions compared against each other. Comparison of warm and cold conditions found a significant loss in expression of *scxa, mkxa, tnmd, thbs4b*, and *sox9a* with warm dissociation (Figure 3), while the zinc-finger transcription factor *egr1* was upregulated with warm dissociation (Figure 3). We also noticed significant upregulation of TGFβ signal transduction genes such as *smad3a, tgif1*, and *tgfb1a* in the warm condition (Figure 4A, Figure 5A), suggesting that cells enter a stressed, TGFβ-dependent pro-inflammatory/pro-fibrotic state.

**Collagenase dissociation at warm temperatures elevates stress responses in embryonic tenocytes**

To try and define specific vs. general effects of warm collagenase dissociation on the tenocyte transcriptome, we identified genes either upregulated or downregulated in warm vs. cold conditions using pseudo-bulk differential expression analysis and compared the results with similar studies in other cell types. Overall, 716 genes were upregulated in warm conditions and 2,142 were upregulated in cold conditions with adjusted p-values < 0.05 (Figure 4A). Gene ontology (GO) analyses of the warm conditions gene list for biological process (BP) terms using clusterProfiler [16] revealed enrichment for terms associated with cell death, cell cycle, and translation (Figure 4B). This suggests that warm dissociation disrupts tenocyte homeostasis prior to sequencing, which may interfere with cellular functions including transcription. Top differentially expressed genes (DEG) sorted by log2-fold change included zebrafish orthologues of Fos, Socs3, and Jun, which are known cell-stress markers previously reported as highly expressed by tumor cells dissociated under warm but not cold conditions [3] (Figure 4C). Similar stress responses occur in mouse kidney cells dissociated with warm but not cold conditions [2], in mouse skeletal muscle cells after injury [20], and in mouse muscle satellite cells as well as zebrafish fin osteoblasts in response to tissue dissociation [1]. Overall, our DEG set overlapped with over 18% of the genes induced in human tumor cells by collagenase and warm conditions [3] (Figure 4D; see methods), and ~50%–60% of the stress-responsive genes induced in muscle satellite cells [1] (dissociated at 37 °C) and osteoblasts [1] (dissociated at 30 °C) (Figure 4D). Additionally, our DEG set significantly overlapped with warm upregulated genes from a study comparing mouse kidney cell 6 °C dissociation with 37 °C dissociation at 15 min and 60 min, respectively, with more substantial overlap with the 60 min dissociation gene set [2] (Figure 4D). Conversely, comparison of the cold upregulated DEGs against these datasets revealed no significant overlap suggesting upregulation of cell stress markers primarily in warm conditions alone [1–3] (Figure 5B). These results suggest that warm collagenase dissociation of tenocytes causes a global tissue-agnostic stress response that is minimized by dissociation under cold conditions.

**Cranial tenocytes downregulate critical ECM genes in warm dissociation conditions**

**Figure 6.**
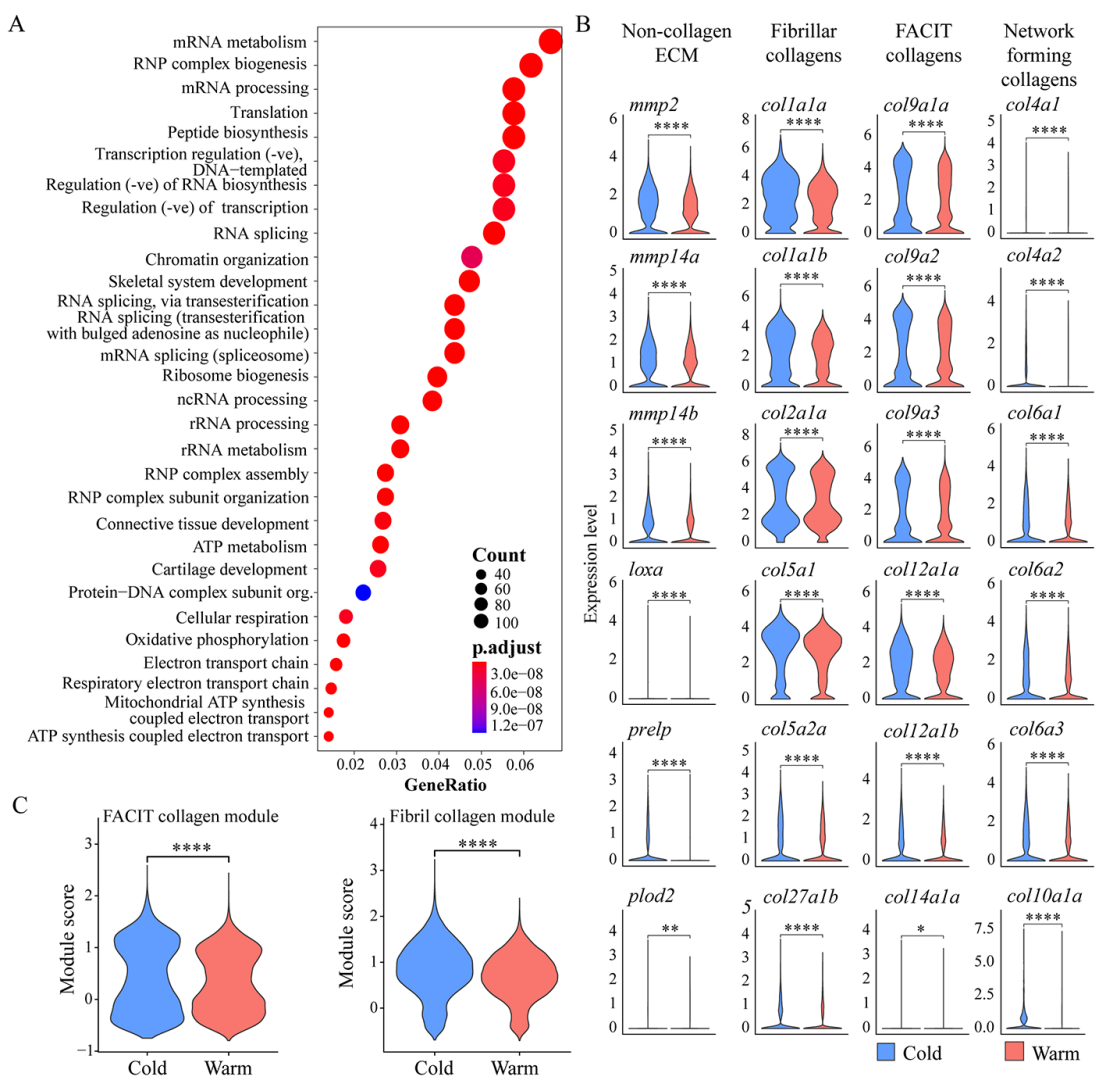
Warm dissociation conditions cause downregulation of ECM genes. (A) Dot plot shows gene ontology (GO) term analysis for biological process (BP) terms of all significant warm-downregulated genes. (B) Violin plots show expression of selected tendon ECM genes between warm vs. cold dissociation conditions, categorized into categories: non-collagen ECM, fibrillar collagens, FACIT collagens, and network-forming collagens. (C) Violin plots showing aggregate module expression score for fibril forming (top) and FACIT (bottom) collagens split by dissociation condition. ns = not significant, * p < 0.05, ** p < 0.01, *** p < 0.001, **** p < 0.0001.

We next examined if tenocyte dissociation under cold conditions preserves in vivo gene expression patterns. Tenocytes secrete the collagens and other ECM proteins that give tendons their fibrillar architecture and respond to signals from the surrounding matrix. Our pseudo-bulk RNA-seq analyses, combining scRNA-seq data from cells dissociated using cold vs. warm conditions, showed that among the top DEGs downregulated in warm conditions by fold change were known tendon/connective tissue markers such as *scxa*, *tnmd*, and *osr1*, and various collagens enriched in tendons (Figure 4A). GO term analysis showed enrichment for BP terms related to skeletal and connective tissue development (Figure 6A). Based on these data, we hypothesized that warm dissociation dysregulates/inhibits the expression of critical tendon ECM genes. To test this, we first focused on global differences in collagen expression between warm and cold conditions. We observed downregulation of a subset of fibrillar collagens (e.g., Col1, 2, 5, and 27 orthologues), fibril-associated collagens with interrupted triple helices (FACIT) collagens (Col 9, 12, and 14 orthologues), and network-forming collagens (Col4, 6, and 10 orthologues) under warm dissociation conditions [21,22] (Figure 6B). When comparing aggregate expression between conditions using gene modules for fibrillar and FACIT collagens, we observed global downregulation with warm dissociation for both (Figure 6C, see Methods). Genes encoding proteins involved in collagen crosslinking, stabilization, remodeling, and degradation were also downregulated in warm dissociation conditions, notably matrix metalloproteinases (MMPs) *mmp14a* and *mmp14b*, lysyl oxidases/hydroxylases *loxa* and *plod2*, and the leucine-rich repeat protein *prelp* [23–28] (Figure 6B). Thus, traditional warm dissociation with collagenase results in global downregulation of a large variety of collagen and non-collagen ECM genes in tenocytes, likely disrupting tenocyte function and thereby skewing interpretations of cell states.

Given the dramatic increase in the use of sequencing technologies to define cell heterogeneity and gene regulatory networks, it is critical to understand how initial tissue sample preparation influences downstream data quality and interpretation [10–12,29,30]. This includes not only the temperature and enzymes used for tissue dissociation but also any addition of transcriptional inhibitors, ribonucleotide analogs, or further isolation of individual cell nuclei [31–33]. Our study, using cold-activated protease dissociation of tendon cells, highlights the benefits of this approach for preserving transcriptional cell states of cells isolated from whole organisms and further establishes its effectiveness in profiling cells from connective tissues with minimal stress.

## General notes and troubleshooting

**General notes**

1. When applying this protocol to dissociate older larvae or larger tissue sections, the volume of protease solution can be doubled. In this protocol, we have used 30 mg of tissue.

2. The working protease solution should be clear without any precipitate.

3. If dissociation fails to proceed after a few minutes, protease (50 μL) can be added to the reaction. If a frozen aliquot fails to show signs of activity (dissociation) within the first 10 min, use freshly prepared enzyme aliquots.

4. If the dissociation mixture is viscous, DNase (10 μL) can be added and incubated on ice for 10 min.

5. If, after washing, the pellet is not completely suspended in DPBS and remains viscous, DNAse (10 μL) can be added to prevent cells from sticking to each other.

6. If the subsequent steps in the protocol involve cell culture, culture media can be used to dilute the cell suspension after filtering through the cell strainers.

**Troubleshooting**

Problem 1: Tissue fails to dissociate.

Possible cause: Protease activity is very low in the mixture.

Solution: Prepare a fresh aliquot of protease.

Problem 2: Low number of cells in the cell suspension.

Possible cause: Cells were lost due to viscous clumps and incomplete dissociation.

Solution: Verify complete tissue dissociation by confirming the absence of tissue clumps using a stereomicroscope. Before filtering through the 40 μm cell strainer, add additional DNAse (10 μL) solution and incubate on ice for 10 min. Wash the cell strainer with 9 mL of DPBS.
